# Editorial: *Helicobacter pylori* infection: pathogenesis, antibiotic resistance, advances and therapy, new treatment strategies

**DOI:** 10.3389/fmicb.2022.1102144

**Published:** 2022-12-08

**Authors:** Maria Teresa Mascellino, Stefano Pontone, Alba Edith Vega, Peter Malfertheiner

**Affiliations:** ^1^Department of Public Health and Infectious Diseases, Sapienza University, Rome, Italy; ^2^Department of Surgical Sciences, Sapienza University, Rome, Italy; ^3^Faculty of Biochemical Chemistry and Pharmacy, National University of San Luis, San Luis, Argentina; ^4^Department of Gastroenterology, Hepatology and Infectious Diseases, Otto-v-Guericke-University, Magdeburg, Germany

**Keywords:** *Helicobacter pylori* infection, molecular diagnostics, antibiotics resistance, non-traditional therapies, updated treatment strategies

*Helicobacter pylori* (*Hp*) is a microorganism discovered only 40 years ago but since then its importance has grown in many pathologies as chronic gastritis, peptic ulcer, gastric carcinoma, and mucosa-associated lymphoid tissue (MALT) lymphoma, as well as other endothelial dysfunctions leading to vascular diseases (Ando et al., 2006). Gastric cancer mainly represents the fifth cancer for incidence and the third cause of death in the developed countries (Rawla and Barsouk, 2019). Epidemiology, transmission, and pathogenesis of *Hp* have been deeply examined as well as the resistance to antibiotics (Eusebi et al., 2014; Kayali et al., 2018; Mascellino et al., 2020). The development of new drugs capable of eradicating this pathogen is highly recommended such as combination therapy or research into other alternatives and new strategies (Makipour and Friedenberg, 2011; Yuan et al., 2021).

The influence of previous therapies in *Hp* eradication rate is reported in the paper by Choe et al. in which the past use of metronidazole (MZ) is taken into consideration in a group of subjects hospitalized in Korea. It was noted that in patients who underwent the BQT (Bismuth Quadruple Therapy) for 14 days and in those with no MZ previous treatment, the eradication rate was higher than in subjects who underwent the BQT for less than 14 days or in patients with MZ previous use.

The susceptibility of *Hp* to antibiotics in the different niches of the stomach (antrum and corpus) is another issue based on the microorganism heteroresistance. The authors Goni et al. take into account the severity of atrophic gastritis from the antrum and corpus biopsies, where *H. pylori* strains were isolated and tested for antibiotics susceptibility. The severity of atrophy seemed to be correlated with the increase in MZ and clarithromycin (CLA) resistance. Thus, the severity of atrophy was a crucial element in order to establish a correct treatment. The high CLA and MZ resistance in atrophic gastritis should be carefully considered.

As far as antibiotic resistance is concerned, interesting is the article by Guo et al. in which the genetic methods (polymerase chain reaction, whole genome sequencing) and the phenotypic method (broth micro-dilution) were compared in *H. pylori*-infected patients with treatment failure for at least twice in order to assess the efficacy of different antimicrobial resistance–guided quadruple therapies in refractory *H. pylori* infection. As such, the genotypic resistance determined using gastric biopsy specimens correlated well with the phenotypic resistance both achieving high eradication rates.

The question of whether susceptibility testing is essential in guiding therapeutic strategies has been debated in many studies (Gomollon et al., 2000; Zullo et al., 2003; Graham, 2015).

In this Research Topic, two articles play an important role for this purpose. The first one by Nyssen et al. deals with the comparison of empirical vs. susceptibility-guided *Hp* treatment. The authors infer that the benefit of susceptibility-guided treatment over empirical therapy could not be demonstrated, even in first-line therapy if the most updated quadruple regimen (BQT) is prescribed.

In the same way in the article by Li P. et al., both susceptibility-guided therapy based on the resistance of CLA or minocycline and empiric quadruple therapy containing furazolidone may achieve the same level of eradication. The empirical quadruple therapy containing furazolidone, bismuth, and esomeprazole might be selected as a correct first-line regimen.

A series of articles in this Research Topic is based on the research for alternative products different from antibiotics and able to treat *Hp* infection (Ayala et al., 2014; Gopal et al., 2014; Ruggiero, 2014).

For example, Sosa et al. found that new therapies based on plant extracts such as extra virgin olive oil show an effect *in vitro* on *Hp* strains and *in vivo* on the gastric mucosa of mice infected orally with an *H. pylori* suspension, greatly preventing the formation of the stomach erosions after the treatment.

The same situation is found in the paper of Ibáñez et al. that examines the *Hp* antimicrobial activity of *Asclepias curassavica* L. a derivative by a plant from South America and Tropical areas, which was considered a therapeutic adjuvant and a safe nutraceutical product. Asclepain showed an interesting activity towards *Hp* even against the drug-resistant isolates. The MIC resulted as being 1–2 μg/ml and the MBC between 2 and 4 μg/ml without toxic effects. Its activity was based on the reduction of *H*p virulence genes such as *ureA*, ompA, and *flaA*.

The following three articles of this Research Topic concern other compounds included in the group of non-antibiotic substances showing an antimicrobial activity. The first study (Jia et al.) takes into consideration the Jinghua Weikang Capsule (JWC) that is the first patent medicine approved in China for the treatment of gastritis and peptic ulcers caused by *Hp*. Its major component *Chenopodium ambrosioides* L. inhibits biofilm formation even though the exact mechanism of its efficacy against drug-resistant *Hp* is still uncertain. It also seems to be able to induce the reversal of MZ resistance.

The second study concerns the use of 1,4-dihydropyridine (DHP)-based antihypertensive drug, which seems to exhibit a strong bactericidal activity against *H. pylori*. The results presented in this study by González et al. strongly support the use of 1,4-DHP as a tool for novel antimicrobials against *H. pylori*. The MIC values are reported to be comparable with those achieved by first-line antibiotics.

In the third study, Li R.-J. et al. selected from the natural product forsythia, the Phillygenin, an effective antibacterial component against *Hp* even if the values of MICs and MBCs are shown to be quite high (16 μg/ml). It was found to be non-toxic to gastric epithelial cells and its mechanism of action was mainly associated with the inhibition of biofilm formation. Phillygenin could also cause ATP leakage in a concentration and time-dependent way. This mechanism seemed to be multiple targets.

In the context of products that can be suitable for helping to combat *Hp* infection either alone or associated with antibiotics, probiotics play a crucial role. They might interact with the gastric microbiota bringing benefits in the clinical *Hp* management. These concepts are discussed in the last article by Marinelli et al. who studied *Lactococcus rhamnosum (LG)* supplementation in combination with BQT (Bismuth Quadruple Therapy) to determine a possible improvement in eradication rate, tolerability, and compliance. The authors found that the influence of LG to BQT in the management of *Hp*-related infection was very useful in terms of efficacy and tolerability but mainly in the persistence decrease of post-treatment dyspepsia.

In conclusion, *Hp* antibiotic resistance has been increasing all over the world in recent years, and this phenomenon constitutes an important challenge for the treatment of this fastidious bacterium (Figure 1). This has led to an obstinate search for new solutions such as treatments based on the use of natural resources such as plants, probiotics, nutraceuticals, and bacteriophages. As such, some interesting non-traditional therapies have been indicated in this Research Topic as a mean to target this important gastric pathogen. Notably, it was also shown in this study that successful *Hp* eradication might be achieved in almost all patients even without susceptibility tests that are expensive and time-consuming.

**Figure 1:**
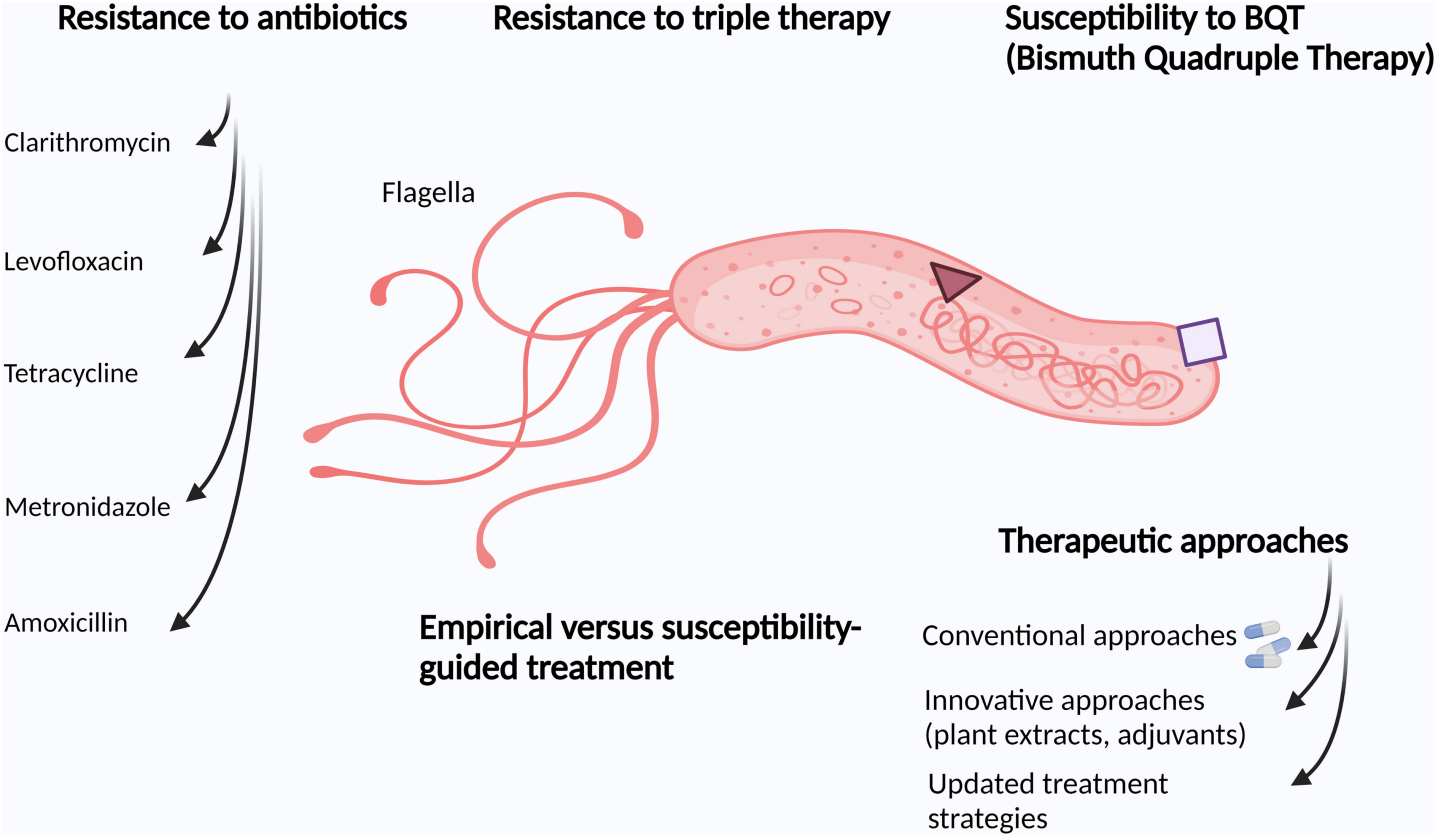
Overview of different features of *Helicobacter pylori* management.

## Author contributions

MM organized the editorial and wrote the paper. SP revised the manuscript. AV checked the references. PM gave a complete overview of the whole article. All authors contributed to the article and approved the submitted version.
